# Supposed pituitary-production of human chorionic Gonadotropin induced by androgen deprivation therapy

**DOI:** 10.1590/S1677-5538.IBJU.2017.0654

**Published:** 2019

**Authors:** Koji Yoshimura, Yoshiharu Nakashima, Kyohei Sugiyama, Naoki Kohei, Akitoshi Takizawa

**Affiliations:** 1 Department of Urology, Shizuoka General Hospital, Shizuoka, Japan;; 2 Department of Urology, International Goodwill Hospital, Yokohama, Japan

**Keywords:** Chorionic Gonadotropin, Neoplasms, Germ Cell and Embryonal, Androgens, Luteinizing Hormone

## Abstract

**Introduction:**

The main cause of slightly elevated human chorionic gonadotropin (HCG) after successful treatment of male germ cell tumors is considered to be pituitary-derived HCG. It is well known that pituitary-derived HCG is frequently detected in postmenopausal women. We evaluated the status of serum HCG in men with elevated gonadotropins, which were induced by androgen deprivation therapy, using commercially available assays.

**Materials and Methods:**

We enrolled 44 patients with prostate cancer, who underwent luteinizing-hormone releasing hormone agonist treatment. We measured serum follicle-stimulating hormone (FSH), serum luteinizing hormone (LH), serum total HCG, serum free HCG*-*β subunit, and urine total HCG 3 times per patient, on the day of treatment initiation, the next day, and 3 months after.

**Results:**

On the day after treatment initiation, serum and urine HCG was detected in 61% and 73% of patients, respectively. Markedly strong correlations were observed between serum/urine HCG and FSH/LH. In particular, receiver operating characteristic curve analysis indicated excellent area under the curve (0.977, 95% confidence interval 0.951–1.003)) for serum HCG-detectable LH. At the cutoff value of 21.07 mIU/mL for serum HCG-detectable LH, the sensitivity and specificity were 96.7% and 95.3%, respectively. Serum HCG-β was not detectable at any times in any patients.

**Conclusions:**

Suggested pituitary-derived HCG can be frequently detected in patients with elevated gonadotropins, and there is a firm association between HCG detection and gonadotropin levels.

## INTRODUCTION

Human chorionic gonadotropin (HCG) is a reliable serum biomarker for determining prognosis and monitoring treatment of germ cell tumors (GCTs). However, slight increases of serum HCG level without viable tumor cells are occasionally observed and misinterpreted as a relapse, leading to unnecessary continuation of chemotherapy. Although there are several categories of unexplained low level HCG after male GCT treatment, most cases have no active disease (1). In patients with GCT, treatment (orchiectomy and chemotherapy) can cause hypogonadism, which induces increased level of luteinizing hormone (LH) and HCG production from the pituitary gland (2).

Since HCG is more frequently used for diagnosing and monitoring pregnancy and trophoblastic disease than for diagnosing and monitoring GCTs, the situations of detectable serum HCG are more extensively investigated in women than in men. It is known that HCG concentrations rise in perimenopausal and postmenopausal women, and the detected HCG is generated from the pituitary gland (3-5). Cole et al. reported that all women, who were consulted for elevated HCG to the USA hCG Reference Service and diagnosed to have no trophoblastic neoplasms or nontrophoblastic tumors, had variably elevated luteinizing hormone (LH) (5). In this study, coefficient of determination (r2) between measures of HCG and LH was reported to be as high as 0.78. However, information on pituitary HCG in men is very limited.

Older men have also higher concentration of HCG than younger men, but the degree of elevation is lower than in postmenopausal women and the elevated HCG concentration in older men can be detected only by very sensitive assays (6). These previous studies suggest that slight LH elevation in older men, which is not extensive like menopausal women, would result in slight HCG elevation, which cannot be detected by commercially available kits. Long-term survivors after GCT chemotherapy have elevated LH according to treatment intensity like older men (7). Since the degree of LH elevation in long-term survivors is not extensive, detectable HCG level would be rare, if any. However, patients currently undergoing chemotherapy could have more elevation of LH than long-term survivors, and extensive elevation of LH might induce detectable HCG level.

We hypothesized that phantom HCG detection without recurrence of GCT is associated with LH elevation. In other words, in men, the situation which accompanies elevation of LH would induce detectable serum HCG, which is produced by the pituitary. The preferred method to examine this effect is a study to measure hormone levels in GCT patients currently receiving chemotherapy. However, the fact that detected HCG could potentially originate from viable malignant cells presents an ethical problem for such a study. Therefore, we selected prostate cancer patients for this study, although their hormonal milieu is different from that of young GCT patients and their eligibility is suboptimal.

Luteinizing-hormone releasing hormone (LHRH) analogue, which is widely used for prostate cancer treatment, induces a large increase in LH and follicle-stimulating hormone (FSH) levels, like after menopause in women, during the first few days after initial administration (8), and it is unknown that old male patients who undergo LHRH analogue treatment have detectable serum HCG like postmenopausal women.

In this study, we attempted first to gather fundamental information regarding HCG detection associated with elevated LH/FSH, which is induced by androgen deprivation therapy (ADT) for prostate cancer. Our second purpose was to elucidate the association between the levels of HCG and gonadotropins. Finally, we attempted to evaluate the impact of renal function on metabolism of HCG, suggested by comparison of serum and urine HCG values.

## MATERIALS AND METHODS

### Patients and treatments

This study was approved by the Ethics Committee, Shizuoka General Hospital.

Entry criteria were: (1) male patients aged 40-80 years; (2) pathologically proven prostate cancer without distant metastasis; (3) no other active malignant disease; and (4) initiation of LHRH agonist (leuprorelin acetate or goserelin acetate) treatment. Between January 2015 and July 2016, 44 consecutive patients were prospectively enrolled. We measured serum and urine hormone levels, as listed below, on the day of initiation of LHRH agonist (T1), on the day after treatment initiation (T2), and after 3 months (T3). At T1, we also measured serum creatinine to assess renal function. Estimated glomerular filtration rate (eGFR) was calculated using Japanese version of Modification of Diet and Renal Diseases equation.

The hormones measured were serum testosterone, serum LH, serum FSH, serum total HCG, serum free HCG-β subunit, and urine total HCG. We measured all these hormones at all 3 measurement times, except for serum testosterone at T2, with assistance of SRL Inc. (Tokyo, Japan). We used commercially available kits for total HCG and HCG-β subunit measurement, that is, Immulyze HCG III (normal range <1.0 mIU/mL; Siemens Healthcare, Tokyo, Japan) for total HCG and Ball Elsa F-βHCG (normal range <0.1 ng/mL; Sceti Medical Labo, Tokyo, Japan) for HCG-β subunit. Ball Elsa F-βHCG kit can detect free HCG-β and nicked free HCG-β subunits, whereas Siemens Immulyze HCG III kit can detect all forms of HCG and HCG degradation products including β-core fragments. Siemens Immulyze HCG III kit shows no cross-reactivity to 16.5 ng/mL (77.385 mIU/mL) concentration of LH, 26.8 ng/mL (513.6 mIU/mL) concentration of FSH, or 860 ng/mL (4.24 mIU/mL) concentration of thyroid stimulating hormone. We used Elecsys Testosterone II (Roche Diagnostic GmbH, Mannheim, Germany) for testosterone, ARCHITECT LH II (Abbott Japan, Matsudo, Japan) for LH, and ARCHTECT FSH (Abbott Japan, Matsudo, Japan) for FSH, respectively.

### Statistical analyses

The correlations of total serum or urine HCG with FSH and LH were analyzed by Spearman's rank correlation coefficient. Comparison between any two patient groups was analyzed with Mann–Whitney's U test. Associations between detection of serum or urine HCG and FSH/LH were evaluated with receiver operating characteristic (ROC) curves. p<0.05 was considered statistically significant.

## RESULTS

### Patient Characteristics

Median age was 71.5 years (range 64-79), and prostate specific antigen (normal range <4 ng/mL) was 10.64 ng/mL (range 0.49-228). Gleason's score was 6 in 4 patients, 7 in 22, 8 in 17, and 9 in 1, and no patients had atypical pathology, such as neuroendocrine differentiation. Three patients had clinically positive lymph nodes.

Twenty-five and nineteen patients underwent leuprorelin acetate and goserelin acetate, respectively. The levels of FSH and LH were highest at T2. The degree of alteration of LH was more marked than that of FSH. The transition of hormone values is shown in Table-1.

**Table 1:** Transition of Hormone Values.

		T1	T2	T3
Testosterone	(ng/mL)	1.98-10.9 (4.58)	--	<0.03-2.27 (0.04)
FSH	(mIU/mL)	2.92-59.71 (12.15)	6.46-95.38 (25.14)	0.77-9.47 (3.40)
LH	(mIU/mL)	2.05-27.82 (6.07)	7.93-110.60 (32.65)	<1.0-2.19 (0.13)
Serum HCG	(mIU/mL)	<1.0-4.6 (<1.0)	<1.0-10.7 (1.4)	<1.0-7.8 (<1.0)
Urine HCG	(mIU/mL)	<1.0-<1.0 (<1.0)	<1.0-7.8 (<1.0)	<1.0-5.0 (<1.0)

The values are shown as ranges with median values in parentheses.

**T1** = baseline; **T2** = one day after treatment initiation; **T3** = 3 months after treatment initiation; **FSH** = follicle-stimulating hormone; **LH** = luteinizing hormone; **HCG** = human chorionic gonadotropin

### Detection of HCG

Of the 44 patients, 2 (5%) had detectable serum HCG (2.1 and 4.6 mIU/mL, respectively) at T1. However, 19 patients (43%) had detectable urine HCG (up to 7.8 mIU/mL) at T1. At T2, 27 (61%) and 32 (73%) patients had detectable serum and urine HCG, up to 10.7 and 7.8 mIU/mL, respectively. At T3, the rates of HCG detection decreased again to 0% (serum) and 11% (urine).

Serum HCG-β was not detectable at any times in any patients.

### Associations between HCG and Gonadotropins

**Figure 1:**
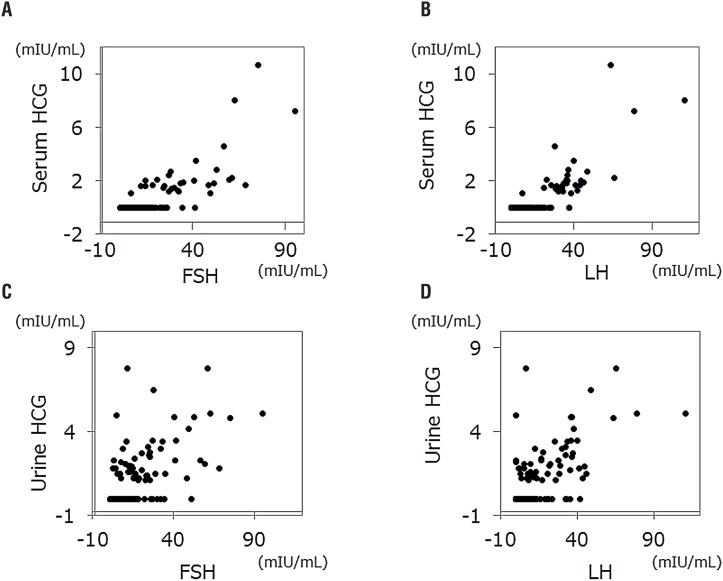
Dot plots between serum gonadotropins and HCG. A) serum HCG versus FSH; B) serum HCG versus LH; C) urine HCG versus FSH; D) urine HCG versus LH.

We analyzed 132 pairs of HCG and FSH/LH values, which were obtained 3 times per patient. Generally, associations between serum/urine HCG and gonadotropins were strong; p<0.001 with correlation coefficient 0.665 between serum HCG and FSH; p<0.001 with correlation coefficient 0.698 between serum HCG and LH; p<0.001 with correlation coefficient 0.605 between urine HCG and FSH; and p<0.001 with correlation coefficient 0.609 between urine HCG and LH (Figure-1).

**Figure 2:**
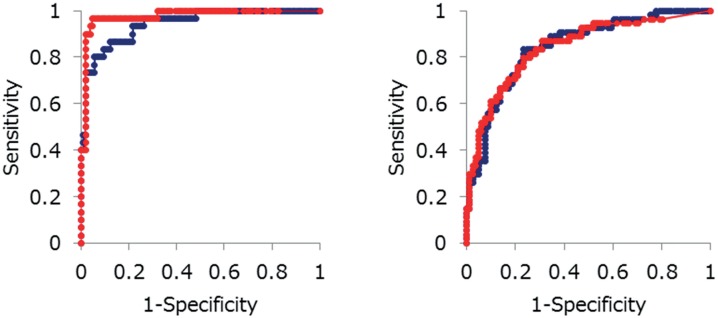
ROC curves for detection of HCG. **A)** Detection of serum HCG; **B)** detection of urine HCG. Red circles and lines indicate LH, and blue circles and lines indicate FSH.

ROC curve analysis indicated that LH and FSH were strong predictors for HCG detection with excellent AUCs; 0.977 (95% CI 0.951-1.003) for serum HCG-detectable LH, 0.944 (95% CI 0.901–0.980) for serum HCG-detectable FSH, 0.843 (95%CI 0.773–0.913) for urine HCG-detectable LH, and 0.845 (95% CI 0.778-0.912) for urine HCG-detectable FSH. At the cutoff value of 21.07 mIU/mL for serum HCG-detectable LH, the optimal sensitivity and specificity were 96.7% and 95.3%, respectively. At the cutoff value of 18.30 mIU/mL for serum HCG-detectable FSH, the optimal sensitivity and specificity were 89.7% and 88.5%, respectively. At the cutoff value of 7.17 mIU/mL for urine HCG-detectable LH, the optimal sensitivity and specificity were 81.5% and 76.6%, respectively. At the cutoff value of 10.32 mIU/mL for urine HCG-detectable FSH, the optimal sensitivity and specificity were 83.3% and 76.9%, respectively (Figure-2).

### Renal Function and Detection of Serum and Urine HCG at T2

At T2, 7 patients (16%) had negative serum and negative urine HCG; 5 patients (11%) had positive serum and negative urine HCG; 10 patients (23%) had negative serum and positive urine HCG; and 22 patients (50%) had positive serum and positive urine HCG. The 10 patients with negative serum and positive urine HCG had significantly higher eGFR than the other patients (p=0.018, 75.8±11.0 versus 63.5±13.2 mL/min).

## DISCUSSION

This study showed that many of old patients with prostate cancer have detectable serum and urine HCG levels when gonadotropins elevate like menopausal women. Our observations may serve as a reference to interpret questionable HCG after treatment of GCT, although they cannot be applied to young patients straightaway. While long-term survivors after GCT chemotherapy have elevated gonadotropins according to treatment intensity (7), patients currently undergoing chemotherapy could have more elevation of gonadotropins like our patients.

Historically, early HCG radioimmunoassays exhibited some cross-reaction with LH and its β subunit. However, neither LH nor LH β subunit is currently a problem with 2-site immunometric assays, which use 2 different antibodies that are directed against different sites on HCG or HCG β subunit (9). Pituitary-derived HCG is considered one of the main causes of unexplained low-level HCG after GCT treatment. Another possible cause is heterophile antibody, such as human anti-mouse antibodies (10). The two assays used in this study may also have been influenced by heterophile antibodies. Antibodies are filtered by the glomeruli, thus, heterophile antibody interference is seldom present in urine samples (11). Therefore, the combination of positive serum HCG and negative urine HCG can result from heterophile antibodies. Four of our patients had positive serum and negative urine HCG at T2. However, none of these patients had positive serum HCG at T1 or T3. Thus, the observation of these 4 patients at T2 was not affected by heterophile antibody. Mass spectrometry would be another good option to rule out phantom HCG due to heterophile antibodies. Free HCG-β subunit, which is not combined with α subunit, is exclusively secreted from malignant cells (12). Free HCG-β subunit was not detected at all, which means that the origin of HCG in our study was not tumor cells. These observations suggest that detected HCG in our study would be derived from the pituitary gland, which could be proved only by immunohistochemistry or RNA assessment for HCG of the pituitary gland.

In this study, the rates of positive serum HCG were high at T2, and no patient had positive serum HCG at T3. Although it has already been reported that healthy men have detectable levels of pituitary-derived HCG using an ultrasensitive assay (4, 6), our observations definitively show that most men with elevated gonadotropins have detectable serum HCG, even with commercially available kits. Although we did not assess this, surgical castration can lead to detectable serum HCG as well as medical castration by LHRH analogue. ROC curve analysis showed a surprisingly strong association between serum HCG detection and serum LH value, indicating the optimal cutoff LH level of 21.07 mIU/mL. Although these results were profoundly dependent on the data of T2, the results analyzed only by the data of T1 and T3 reveal almost the same results (data not shown). This observation suggests that LH level <21 mIU/mL can definitely rule out detectable pituitary HCG. However, LH level >21 mIU/mL does not always mean that questionable HCG originated from the pituitary gland, because the combination of high LH level and detectable HCG derived from active tumor is possible. If detected HCG after GCT treatment is questionable, testosterone-loading test would be an examination of choice (2, 13).

Urine HCG was also strongly associated with elevated gonadotropins. As correlation coefficients and ROC curves indicated, however, this association was weaker than the association between serum HCG and gonadotropins. These findings suggest that several factors other than gonadotropins affect detection of urine HCG. One of the possible factors is renal function.

HCG is degraded and excreted by the kidneys, resulting in detection of HCG and its degradation products in urine (14). Thus, end-stage renal diseases, in which metabolic clearance of HCG decreases, can cause abnormal elevation of serum HCG level (15, 16). Conversely, good renal function can accelerate excretion of HCG, leading to lower serum HCG level. In our study, renal function of patients with negative serum and positive urine HCG was significantly better than that of the other patients. This observation is compatible with the aforementioned hypothesis. Therefore, it might be necessary for us to pay attention to renal function when interpreting values of serum and urine HCG.

There are several limitations to this study. First, the study population was small. Second, the patients were not completely healthy and had prostate cancer. Although there have been no studies reporting HCG secretion by prostate cancer cells, the interaction between prostate cancer cells and serum HCG may not be completely denied. Third, our patients were older than typical male GCT patients; therefore, our results cannot be applied to young patients straightaway. Fourth, it was not definitely proven that HCG measured in our study was derived from the pituitary gland, despite circumstantial evidence. Finally, we used only 2 kinds of assays to measure HCG. If we used other types of assays, the results may be different. Despite all of these limitations, we believe that this study could be a cornerstone of further studies regarding pituitary-derived HCG in men.

## ABBREVIATIONS

ADT: = androgen deprivation therapy
AUC: = area under the curve
eGFR: = estimated glomerular filtration rate
FSH: = follicle-stimulating hormone
GCT: = germ cell tumor
HCG: = human chorionic gonadotropin
LH: = luteinizing hormone
LHRH: = luteinizing-hormone releasing hormone
ROC: = receiver operating characteristic
